# The Value of Lactate Dehydrogenase in Predicting Rhabdomyolysis-Induced Acute Renal Failure; a Narrative Review

**DOI:** 10.22037/aaem.v9i1.1096

**Published:** 2021-03-09

**Authors:** Hazhir Heidari Beigvand, Kamran Heidari, Behrooz Hashemi, Amin Saberinia

**Affiliations:** 1 Preventive Medicine and Public Health Research Center, Psychosocial Health Research Institute, Community and Family Medicine Department, School of Medicine, Iran University of Medical Sciences, Tehran, Iran.; 2 Skull Base Research Center, Loghman Hakim Hospital, Shahid Beheshti University of Medical Sciences, Tehran, Iran.; 3 Emergency Department, Shohadaye Tajrish Hospital, Shahid Beheshti University of Medical Sciences, Tehran, Iran.; 4 Emergency Department, Bahonar Hospital, Faculty of Medicine, Kerman University of Medical Sciences, Kerman, Iran.

**Keywords:** L-Lactate Dehydrogenase, Acute Kidney Injury, Rhabdomyolysis, Role, Review, Clinical Enzyme Tests

## Abstract

**Introduction:**

Determining the diagnostic value of available biomarkers in predicting rhabdomyolysis-induced acute kidney injury (AKI) is a priority. This study aimed to review the current evidence about the value of lactate dehydrogenase (LDH) in this regard.

**Methods:**

In this narrative review, the papers in PubMed, Embase, and web of science were studied. The keywords prognosis, prognoses, prognostic, LDH, rhabdomyolysis, emergency patients, and acute kidney failure or AKI had been selected from MeSH medical dictionary. Related papers written in English and published from November 2007 to December 2020 were selected.

**Results:**

Finally, 14 articles were accepted for analysis. Among the selected articles, four were randomized clinical trials, seven were cross-sectional, and three were case-control studies. The results of the present review showed that abuse of illegal drugs is the most common cause of rhabdomyolysis. AKI is the most serious complication of rhabdomyolysis reported in the studies. These studies have shown a three-fold increase in AKI following drug-induced rhabdomyolysis. The review of the included articles shows that high LDH can predicts AKI, especially in critical and emergency situations such as rhabdomyolysis where there is a risk of death if diagnosed late. These studies show that LDH increases in the presence of renal failure and tissue damage.

**Conclusion:**

Serum LDH is an appropriate and cost-effective prognostic indicator that can be used for risk classification of patients at risk for rhabdomyolysis-induced AKI.

## Introduction

Rhabdomyolysis accounts for 5 to 25% of all acute kidney injuries (AKIs) (1). The overall mortality rate of patients with rhabdomyolysis is about 10% but it is higher in men than women (2). Rhabdomyolysis-induced AKI can occur following poisoning, trauma, environmental factors, metabolic disorders, infections, immune disorders, and hereditary disorders (3, 4). It is also a major medical complication of disasters victims (5). 

Considering the importance of early diagnosis of at-risk patients in emergency department, determining the diagnostic value of available biomarkers in predicting rhabdomyolysis induced AKI is a priority (2, 6).

Muscle damage leads to membrane disruption, extracellular fluid leakage, and increased serum level of creatine phosphokinase (CK) and lactate dehydrogenase (LDH) enzymes (7-10). LDH levels are elevated in infarction, hepatocellular damage, carcinomas, and any conditions that cause cell necrosis (11, 12). 

Various studies have evaluated the level of this enzyme at the time of kidney damage and have shown that in most cases, very high concentrations of LDH have been observed in the serum of patients with rhabdomyolysis, which indicates that it can be useful in predicting renal failure (13, 14). Based on the above-mentioned reasons, this study aimed to review the current evidence regarding the value of LDH in predicting the rhabdomyolysis induction AKI.

## Method:

**Study design**

In this narrative review, the keywords prognosis, prognose, prognostic, lactate dehydrogenase (LDH), rhabdomyolysis, emergency patients, and acute kidney injury or acute renal failure had been selected from MeSH medical dictionary. Related papers written in English and published from November 2007 to December 2020 were searched in PubMed, Embase, and web of science. Original researches with available full text, which had evaluated the value of LDH in detection of at-risk patients for rhabdomyolysis-induced AKI were selected. Case reports, letters to the editors, and review papers were omitted. 

**Search process and assessment of papers**

There was no restriction on duration of intervention, type of participants, and place of study. The steps for selecting the papers were as follows: First the initial search was performed by two researchers separately and the duplicate papers were removed, then with the aim of eliminating unrelated articles, the abstract and the title of each article were reviewed, potentially related papers meeting the inclusion criteria were identified. The full texts of the papers were reviewed by two authors and discussed until consensus was reached regarding their suitability.

After compiling a list of study titles and abstracts in the databases, the Strobe standard checklist was used to determine the quality of the studies. The Strobe checklist consists of 22 different sections that assess various aspects of methodology including sampling methods, statistical analysis, confounding factor adjustment, measurement of variables, validity and reliability of the tools used, and the objectives of the study. 

**Data extraction**

The specific characteristics of each study were extracted by the researchers using a standard form. Two researchers examined the data independently, based on the inclusion criteria. In case of disagreement between the researchers, help was sought from a third researcher.

The differences in findings were resolved using the method of benchmarking the overall conclusions of the articles and in this study, it was organized. Findings on rhabdomyolysis, AKI, and their association with lactate dehydrogenase were derived.

## Results

**Figure 1:**
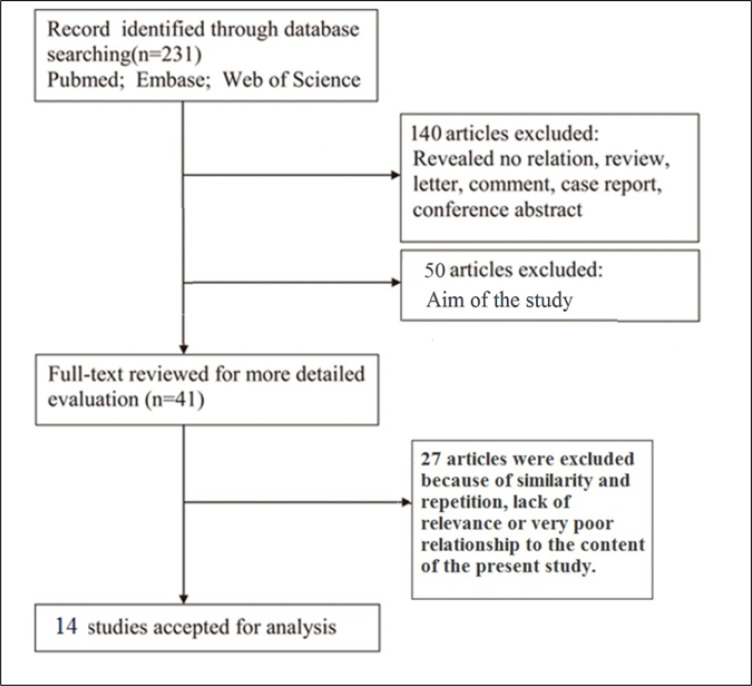
Schematic diagram of article selection process for the present review

231 titles were extracted. In the initial screening stage, the title and type of articles were reviewed and finally 140 articles were excluded. Then, the full text of the articles was studied and 50 articles were excluded due to differences in purpose. Of the remaining 41 articles, 27 articles were excluded due to duplication of content and exclusion criteria, and finally, 14 articles were accepted for analysis (figure 1). The specifications and findings of the included articles are shown in Table 1.

Among the selected articles, four were randomized clinical trials, seven were cross-sectional, and three were case-control studies. The studies, either specifically or generally, examined serum level condition and prognostic ability of LDH in predicting rhabdomyolysis-induced AKI. Amongst the fourteen articles, eight (three randomized clinical trials, two case-control, and three cross-sectional studies) reported a high serum level and prognostic value of LDH in kidney disease. The target groups were often 30-60 year-old individuals and a case-control study was conducted on rats. 

The results of the present review showed that abuse of illegal drugs is the most common cause of rhabdomyolysis. AKI is the most serious complication of rhabdomyolysis reported in the studies (15-18). These studies have shown a three-fold increase in AKI following drug-induced rhabdomyolysis (16). The review of the included articles shows that high LDH can predicts AKI, especially in critical and emergency situations such as rhabdomyolysis where there is a risk of death if diagnosed late (15, 17-23). 

## Discussion

The studies showed that the serum level of LDH was high in patients with renal impairment and the concentration of this enzyme significantly increased in patients with rhabdomyolysis. Studies have also shown that LDH can be useful in early diagnosis of acute renal failure in children. 

The exact process or processes of the development of rhabdomyolysis-induced AKI are not yet clear. A number of proposed processes include mechanical damage to renal tubules caused by myoglobin deposition, and direct effect of the toxic property of free iron on renal tubules and volume depletion (15-18, 20, 21). 

The researchers reported that LDH levels significantly increased in acute renal failure, especially in conditions caused by rhabdomyolysis (15-18). Understanding the cause of this increase can help in providing scientific solutions. Tissue concentration of LDH is 500 times its serum concentration. In general, high concentrations of this enzyme are present in the liver, heart, red blood cells, skeletal muscles, and kidneys (14). In cases of damage to the mentioned organs, such as in kidney and heart infarction and hemolysis, its serum levels rise. Unlike enzymes such as aspartate transaminase (AST), alanine transaminase (ALT), and Creatine phosphokinase (CPK), which show significant variations in enzymatic activity between tissues, for LDH, the difference between the highest value tissues (such as the liver) and the lowest value tissues (such as the kidney) is only about 1.5 times (24). 

In the analysis of the reviewed studies, it can be concluded that serum LDH is an appropriate and cost-effective prognostic indicator that can be used for risk classification of patients with acute renal failure due to rhabdomyolysis. Given the importance of rhabdomyolysis-induced acute renal failure, it is recommended that serum LDH level be measured at the time of admission to the emergency department and in the event of symptoms such as coma, convulsions, and elevated body temperature during the hours after hospitalization in order to promptly start treatment if rhabdomyolysis is proven.

Further understanding of the value of LDH in predicting rhabdomyolysis-induced AKI could be the subject of more extensive research, as present studies in this area are limited to a small number of randomized controlled trials, case studies, and retrospective studies. We need to conduct more multi-centered studies to confirm the findings of the present review and deeply explore the mechanism.

**Table 1:** Characteristics and findings of included studies

Characters*	**Title**	**Findings**
Sanjay et al, 2019 (19). India N = 181 Clinical trial	Epidemiology and outcome of acute kidney injury due to venomous animals from a subtropical region of India	Compared to the survival group, serum white blood cells, serum bilirubin, aspartate aminotransferase, alanine aminotransferase, creatine kinase, lactate dehydrogenase, and serum albumin levels were significantly higher in patients who died. The proportion of patients with leukocytosis, hyperkalemia, metabolic acidosis, pneumonia / ARDS, seizures / encephalopathy, need for ICU support, and dialysis was significantly higher in patients who died.
Akerstrom, et al, 2019 (15). Sweden N = 87 Clinical Trial	rA1M-035, a Physicochemically improved human recombinant α1-microglobulin, has therapeutic effects in rhabdomyolysis-induced acute kidney injury	In this study, the researchers noted that lactate dehydrogenase levels show significant changes in acute renal failure due to rhabdomyolysis.
Qingying et al, 2018 (16). China N = 22 Cross sectional	Clinical analysis in patients with rhabdomyolysis and acute kidney injury caused by intense exercise	There was a significant relationship between elevated serum LDH levels and acute renal failure. Intense exercise in summer is likely to cause rhabdomyolysis and AKI. Early diagnosis and comprehensive treatment including appropriate blood purification are crucial for a successful treatment. The findings also emphasize the importance of age in muscle injury and the monitoring of electrolytes, markers of muscle damage and renal function for prevention of rhabdomyolysis and its related complications.
Hefziba et al, 2017 (17). Israel N = 150 Cross sectional	Serum lactate dehydrogenase is elevated in ischemic acute tubular necrosis but not in acute rejection in kidney transplant patients	A strong and statistically significant association between elevated serum LDH 1 to 3 days before renal biopsy and ischemic ATN diagnosis after kidney transplantation was confirmed.
Akina et al, 2017 (18). Japan N = 59 Case control	Evaluations of lipid peroxidation and inflammation in short‐term glycerol‐induced acute kidney injury in rats	In this study, the researchers stated that there was a statistically significant relationship between time and serum LDH levels and acute renal failure.
Hiroak etal, 2016 (20). Japan N = 86 Cross sectional	Acute Kidney Injury and Rhabdomyolysis After Protobothrops flavoviridis Bite: A Retrospective Survey of 86 Patients in a Tertiary Care Center	Systolic blood pressure, serum creatinine, serum creatine kinase, that serum lactate dehydrogenase white blood cell count, and platelet count differed significantly between the AKI and non-AKI groups (P = 0.01)
Alzahri et al, 2015 (21) Saudi Arabia N = 55 Clinical Trial	Lactate Dehydrogenase as a Biomarker for Early Renal Damage in Patients with Sickle Cell Disease	This study shows that in sickle cell patients LDH correlates with creatinine clearance and, therefore, LDH could serve as a biomarker to predict renal insufficiency in these patients.
Bennett et al, 2015 (22). USA N = 368 Clinical Trial	Pediatric reference ranges for acute kidney injury biomarkers	In this study, the researchers suggested that lactate dehydrogenase marker could be useful in early detection of acute renal failure in children.
Richard et al, 2013 (23). Brazil N = 45 Case control	Renal Cortical Lactate Dehydrogenase: A Useful, Accurate, Quantitative Marker of In Vivo Tubular Injury and Acute Renal Failure	The results indicate that renal cortical LDH assay is a highly accurate quantitative technique for gauging the extent of experimental acute ischemic and toxic renal injury. The fact that it avoids the limitations of more traditional AKI markers implies great potential utility in experimental studies, which require precise quantitation of tubule cell death.
Yue et al, 2013 (13). China N = 192 Cross sectional	Aims to evaluate the prognostic value of D - lactate dehydrogenase in patients with kidney disease	Findings suggest that decreased LDHD expression may be a predictor of poor prognosis in patients with renal failure and even renal cancer.
Weide et al, 2013 (25). Germany N = 855 Cross sectional	Serum markers lactate dehydrogenase and S100B predict independently disease outcome in melanoma patients with distant metastasis	The researchers noted that changes in serum lactate dehydrogenase levels were useful as a marker in the prognosis of soft tissue disease, including kidney disease. In addition, complete metastasectomy has an independent prognosis specifically for the patient subgroup with normal LDH and S100B values.
Gurkan et al, 2010 (26). USA N = 40 Cross sectional	Aims to investigate lactate dehydrogenase as a predictor of kidney involvement in patients with sickle cell anemia	Multivariate analysis revealed a significant correlation between microalbuminuria and LDH level (p = 0.04) when controlled for age, sex, eGFR, hemoglobin level, Fetal hemoglobin%, type of SCA, BMI, history of transfusions, and reticulocyte count. In this pediatric SCA population, LDH was found to correlate with the presence of microalbuminuria and proteinuria. Further studies are needed to confirm LDH as an early marker for the risk of kidney involvement among SCA patients.
Mohammadi-Karakani et al, 2007 (27). Iran N = 57 Case control	Aim of urinary enzymes including LDH as an early marker of kidney injury in diabetic patients	Researchers have shown that LDH urine excretion can be useful in assessing renal failure Patients with diabetes and confirmation of using LDH as a routine screening test.
Huang et al, 2007 (28). Taiwan N = 20 Cross sectional	The objective of the study was to investigate initial clinical characteristics that can suggest an early diagnosis of patients with acute renal infarction presenting with flank and/or abdominal pain in the emergency department (ED).	There was a significant relationship between serum LDH and patient urine and renal infarction. This study, delineated specific clinical features for emergency physicians to raise their suspicion index for an early diagnosis of patients with renal infarction, a disease which is uncommon and is usually delayed or missed at initial ED presentation.

*Characters: authors, year, country, sample size, and type of study, respectively.

ARDS: Acute respiratory distress syndrome; ICU: intensive care unit; LDH: Lactate Dehydrogenase; AKI: Acute kidney injury; LDHD: lactate dehydrogenase D; SCA: sickle cell anemia; BMI: body mass index; eGFR: Estimated Glomerular Filtration Rate; ED: emergency department.

## Conclusion:

The results showed that the level of LDH was high in patients with renal impairment and this enzyme significantly increased in patients with rhabdomyolysis. Studies have also shown that LDH can be useful in early diagnosis of acute renal failure in children. 
